# Hybridization capture reveals microbial diversity missed using current profiling methods

**DOI:** 10.1186/s40168-018-0442-3

**Published:** 2018-03-27

**Authors:** Cyrielle Gasc, Pierre Peyret

**Affiliations:** Université Clermont Auvergne, INRA, UMR 454 MEDIS, 28, place Henri Dunant, F-63000 Clermont-Ferrand, France

**Keywords:** Hybridization capture, Microbial community profiling, 16S rRNA gene

## Abstract

**Background:**

Microorganisms comprise the majority of living organisms on our planet. For many years, exploration of the composition of microbial communities has been performed through the PCR-based study of the small subunit rRNA gene due to its high conservation across the domains of life. The application of this method has resulted in the discovery of many unexpected evolutionary lineages. However, amplicon sequencing is subject to numerous biases, with some taxa being missed, and is limited by the read length of second-generation sequencing platforms, which drastically reduces the phylogenetic resolution.

**Results:**

Here, we describe a hybridization capture strategy that allows the enrichment of 16S rRNA genes from metagenomic samples and enables an exhaustive identification and a complete reconstruction of the biomarker. Applying this approach to a microbial mock community and a soil sample, we demonstrated that hybridization capture is able to reveal greater microbial diversity than 16S rDNA amplicon sequencing and shotgun sequencing. The reconstruction of full-length 16S rRNA genes facilitated the improvement of phylogenetic resolution and the discovery of novel prokaryotic taxa.

**Conclusions:**

Our results demonstrate that hybridization capture can lead to major breakthroughs in our understanding of microbial diversity, overcoming the limitations of conventional 16S rRNA gene studies. If applied to a broad range of environmental samples, this innovative approach could reveal the undescribed diversity of the still underexplored microbial communities and could provide a better understanding of ecosystem function.

**Electronic supplementary material:**

The online version of this article (10.1186/s40168-018-0442-3) contains supplementary material, which is available to authorized users.

## Background

With an estimated total number of 4–6 × 10^30^ cells, prokaryotes are the most diverse and abundant cellular life forms on Earth [1, 2]. With the advent of PCR and high-throughput next-generation sequencing (NGS) technologies, the small subunit rRNA gene has become the most widely used marker for molecular ecology, providing microbial community diversity information in a cultivation-independent manner [3]. PCR-based studies targeting the 16S rRNA gene have led to the discovery of many unexpected evolutionary lineages [4]. However, partial 16S rRNA gene sequences produced from NGS short-read sequencing platforms often result in incorrect or inaccurate taxonomic assignment of amplicons [5] and do not reflect community diversity [6]. Moreover, amplicon sequencing is subject to numerous biases such as differential amplification efficiencies, preferential amplification of specific targets, and unability of degenerate primer to target all the intended targets [6, 7]. Shotgun reads obtained from metagenomic studies provide a source of sequences that are not subject to these major concerns and give access to longer 16S rDNA sequences that enhance phylogenetic assignment [5]. However, because of the short and random nature of metagenomic sequences, most of the informative regions of the 16S rRNA genes might be missed. Indeed, shotgun sequencing of metagenomics samples preferentially provides sequences of dominant microorganisms, thus diminishing the phylogenetic description of microbial communities. Several methodological [8, 9] or bioinformatic [10, 11] strategies have been developed to recover complete or near-complete rRNA genes, but all of them suffer major limitations linked to the difficulties inherent in completely exploring complex microbial diversity.

To overcome the limitations related to microbial communities profiling with conventional molecular approaches, we developed a new hybridization capture method that allows the targeted enrichment of 16S rRNA genes from microbial communities. Gene capture approaches by hybridization traditionally use tiling probes to specifically target and enrich specific biomarkers or genomic regions from genomic DNA isolated from model organisms for resequencing experiments in order to identify new genetic variants [12]. Nevertheless, hybridization capture also allows the capture of divergent targets as it is often done for ancient DNA capture or non-reference species capture. The impact of sequence divergence on the efficiency of hybridization capture has already been evaluated. For example, Hedtke et al. [13] used exome capture to enrich DNA across frog species spanning approximately 250 million years of evolutionary divergence (up to approximately 10% divergence). Thus, hybridization authorizes mismatches between probes and distant targeted sequences not already referred in database. We demonstrated the efficiency of such sequence capture to explore the methanogenic communities present in a lacustrine environment by targeting the methyl coenzyme M reductase subunit A (*mcrA*) gene with a set of nonoverlapping probes, which targeted both known sequences and potential undescribed variants of the *mcrA* gene [14]. Here, we report the first application of a hybridization capture strategy [15] that uses a set of probes targeting all known 16S rRNA gene bacterial and archaeal diversity to enrich the full-length biomarker and to explore the microbial diversity of a metagenomic soil sample contaminated with hexachlorocyclohexane (HCH). To evaluate the efficiency of this strategy, we applied this approach to a microbial mock community and we compared it to 16S rRNA gene amplicon sequencing and shotgun sequencing approaches.

## Methods

### Microbial mock community

An artificial mixture composed of 21 bacterial and 7 archaeal species (representing 6 phyla, 13 classes, 19 orders, 23 families, and 26 genera) whose genome are sequenced was made from genomic DNA extracted from pure cultures of the different species (DSMZ) (Additional file 1: Table S1). Abundances of the different species based on the 16S rDNA copy number per genome and the number of genomes in the mixture was defined so that the final community profile reflects the abundance variability of species in an environmental microbial community (Additional file 1: Table S1).

### DNA isolation from soil

A genomic DNA sample was extracted from a hexachlorocyclohexane (HCH)-contaminated soil sample collected from an old chemical factory (Huningue, France) using a PowerSoil DNA Isolation Kit (MoBio).

### Shotgun sequencing of the samples

Two NGS libraries were constructed from the microbial mock DNA mixture and the soil genomic DNA using the Nextera and TruSeq Kits (Illumina), respectively, according to the manufacturer’s instructions. Libraries were directly sequenced in two paired-end (2 × 250 bp) MiSeq runs (Illumina).

### 16S rRNA gene amplicon sequencing

An approximately 300-bp fragment from the V4 variable region of the 16S rRNA genes was amplified from the microbial mock DNA mixture and the soil genomic DNA by PCR using “universal” primers F515 (GTGCCAGCMGCCGCGGTAA) and R806 (GGACTACHVGGGTWTCTAAT) [16]. Two sequencing libraries were then constructed following the TruSeq DNA library preparation protocol (Illumina), and sequencing of the amplicons was performed on the MiSeq platform (2 × 300 bp, Illumina).

### Hybridization capture targeting the 16S rRNA gene

For probe design, a 16S rDNA-curated sequence database corresponding to all 16S rRNA gene diversity was constructed from the EMBL [17]. A set of 28- to 50-mer degenerate probes (Additional file 1: Table S2) was designed from these databases using KASpOD [18] and PhylArray [19] software that enable the determination of degenerate explorative capture probes. A minimal probe set composed of 15 probes has been selected so that probes are distributed over the entire length of the gene and can hybridize all the known 16S rRNA gene sequences used for probe design and potentially other 16S rDNA variants never described in databases. Probe length has been selected so that probes have a medium size and a GC content (Additional file 1: Table S2) that provides a good compromise between sensitivity and specificity and that allows some mismatches between probes and their targets to enrich all the 16S gene diversity [20]. Adaptor sequences were added to the ends of the probes to enable their amplification by PCR, resulting in “ATCGCACCAGCGTGT-N_X_-CACTGCGGCTCCTCA” sequences, with N_X_ representing the 16S rRNA gene-specific capture probes. Biotinylated RNA capture probes were then synthesized as described by Ribière et al. [21]. In brief, adaptors containing the T7 promoter were added to the 16S rRNA gene-specific capture probes via ligation-mediated PCR, and the final biotinylated RNA probes were obtained after in vitro transcription and purification.

To perform hybridization capture, 2.5 μg of salmon sperm DNA (Ambion) and 500 ng of denatured Illumina library constructed from the microbial mock community or the soil sample were mixed, denatured for 5 min at 95 °C, and incubated for 5 min at 65 °C before adding 13 μl of prewarmed (65 °C) hybridization buffer (10X SSPE, 10X Denhardt’s solution, 10 mM EDTA and 0.2% SDS) and 500 ng of prewarmed (65 °C) biotinylated RNA probes. After hybridization at 65 °C for 24 h, the probe/target heteroduplexes were captured using 500 ng of washed streptavidin-coated paramagnetic beads (Dynabeads M-280 Streptavidin, Invitrogen). The beads were collected using a magnetic stand (Ambion) and washed once at room temperature with 500 μl 1X SSC/0.1% SDS and three times at 65 °C with 500 μl prewarmed 0.1X SSC/0.1% SDS. The captured fragments were eluted with 50 μl 0.1 M NaOH. After magnetic bead collection, the DNA supernatant was transferred to a sterile tube containing 70 μl of 1 M Tris–HCl pH 7.5 and PCR-amplified using primers complementary to the library adapters. To increase the enrichment efficiency, a second round of hybridization capture was performed using the first-round capture products. Enriched DNA from the mock community and the soil sample were then sequenced using two Illumina MiSeq 2 × 250 bp runs.

### Sequencing data processing

#### Microbial mock community

Reads obtained from the shotgun sequencing, amplicon sequencing, and hybridization capture from the microbial DNA mixture were deposited in the NCBI open access sequence read archive (SRA) under accession numbers SRR5381736, SRR5381734, and SRR5381738, respectively. Reads were scanned for library adaptors and quality-filtered using the PRINSEQ-lite PERL script [22] prior to analysis. After filtering, 1,097,064 pairs of reads were obtained from the shotgun sequencing library, 9,259,211 were obtained from the hybridization capture library, and 8,218,732 pairs of reads were obtained from the amplicon sequencing library. For comparison, the sequence number of each sample was randomly normalized to the same sequencing depth, i.e., 1,097,064 paired-end sequences per sample.

The proportions of 16S rRNA gene sequences were estimated in the three datasets using SortMeRNA [23] with the default parameters.

Reads obtained from the three methods were mapped against the 1,922,213 16S rDNA sequences from Silva SSURef 128 database using Bowtie2 (V2.1.0) [24] with end-to-end very sensitive mode. The number of reads aligned in each 16S rDNA sequence from Silva database and the coverage per sequence position were calculated using SAMtools 1.3 [25]. Genera totaling more than 200 mapped reads were considered as present in the samples, which represents an average coverage of 30X over the entire length of the 16S rDNA. Such coverage enables the complete and unambiguous reconstruction of the biomarker.

#### Soil sample

Reads obtained from the shotgun sequencing, amplicon sequencing, and hybridization capture from the soil sample were deposited in the NCBI open access sequence read archive (SRA) under accession numbers SRR3546814, SRR3648004, and SRR3654007, respectively. Reads were scanned for library adaptors and quality-filtered using the PRINSEQ-lite PERL script [22] prior to analysis. After filtering, 19,377,521 pairs of reads were obtained from the shotgun sequencing library, 3,719,256 were obtained from the hybridization capture library, and 529,078 pairs of reads were obtained from the amplicon sequencing library. Sequences of each sample were not randomly normalized to the same sequencing depth. Indeed, the sequencing depth is inversely proportional to the number of 16S rRNA gene sequences expected for each sample: a lower sequencing depth is required for amplicon sequencing where approximately all sequences correspond to the 16S rRNA gene, deep sequencing is necessary for shotgun metagenomics where the biomarker is presumed to represent a very small fraction of the reads, and an intermediate depth is suitable for hybridization capture where a portion of the reads are of interest but still represent the majority of the data compared with shotgun sequencing as a consequence of the targeted enrichment.

The proportions of 16S rRNA gene sequences were estimated in the three datasets using SortMeRNA [23] with the default parameters. 16S rRNA gene reconstruction and OTU clustering from the three samples were performed using EMIRGE 0.60 [11]. Chimeric sequences were eliminated with Uchime 4.2.40 [26] using the ChimeraSlayer “Gold” database using a 0.28 cutoff score. Taxonomic classification of the sequences was then made with RDP Classifier [27] using the Silva [28] database 119 release with a confidence cutoff set at 0.5. We did not correct the microbial abundance considering the 16S copy number variation among taxa because we favored the overall comparison of the three molecular methods rather than the precise description of microbial communities in our soil sample.

Classification of the unassigned 16S rDNA sequences was performed as described by Flandrois et al. [29]. In brief, BLASTN was run on the 16S rDNA sequences against the SSU rDNA stringent database containing 234,263 bacterial and archaeal sequences from GenBank with the expectation value set to 0.1. The 50 sequences with the highest similarity scores were extracted, and multiple alignments were generated against the query sequence using MAFFT [30]. FastTree [31] was then used to reconstruct the tree by approximate maximum likelihood using the general time reversible (GTR) model. Branch support was calculated with the Shimodaira–Hasegawa (SH) test. The 12 novel 16S rDNA sequences described in the present work were deposited in the Genbank database under accession numbers KX363569 to KX363580.

The distance matrixes between the unassigned 16S rDNA sequences and the closest representative sequences identified through phylogenetic placement were performed with Clustal Omega [32]. The thresholds were set at 80, 85, and 90% identity to define the taxonomic phylum, class, and order levels, respectively.

## Results and discussion

We developed a hybridization capture strategy that uses a set of 15 degenerate explorative probes targeting 16S rRNA gene bacterial and archaeal diversity to enrich the full-length biomarker and to explore the microbial diversity of a metagenomic samples (Additional file 1: Figure S1). Enrichment relies on the ability of probes designed on highly conserved regions along the 16S rRNA reference genes to specifically capture DNA from a broad range of species. In the present study, we validated our method on a microbial mock community composed of 28 species and compared it with shotgun sequencing and 16S rRNA gene amplicon sequencing with the widely used F515-R806 primer pair [16]. To demonstrate the efficiency of the method on a real microbial community, we then applied this approach to characterize the microbial structure of a metagenomic soil sample.

### Validation on a microbial mock community

We first applied our hybridization capture method, amplicon sequencing, and shotgun sequencing to a synthetic metagenome composed of DNA extracted from 21 bacterial and 7 archaeal species belonging to 26 phylogenetically distant genera (Additional file 1: Table S1). Relative abundances of the different species have been selected so that the final community profile reflects the species abundance variability in an environmental microbial community, with abundant microorganisms (e.g., *Clostridium acetobutylicum* representing 32.63% of the community) and microorganisms belonging to the rare biosphere (e.g., *Methanobrevibacter smithii* and *Metahnococcus aeolicus*, each accounting for 0.00006% of the community).

Firstly, we calculated the proportion of reads reflecting 16S rRNA genes in the three datasets and demonstrated that hybridization capture leads to a significant enrichment of the 16S rRNA gene. Indeed, 97.30% of reads correspond to the 16S rRNA gene for hybridization capture compared with shotgun sequencing in which only 0.41% of reads carry the phylogenetic biomarker. Thus, hybridization capture enables an optimal enrichment comparable to that obtained with amplicon sequencing (99.52%).

**Fig. 1:**
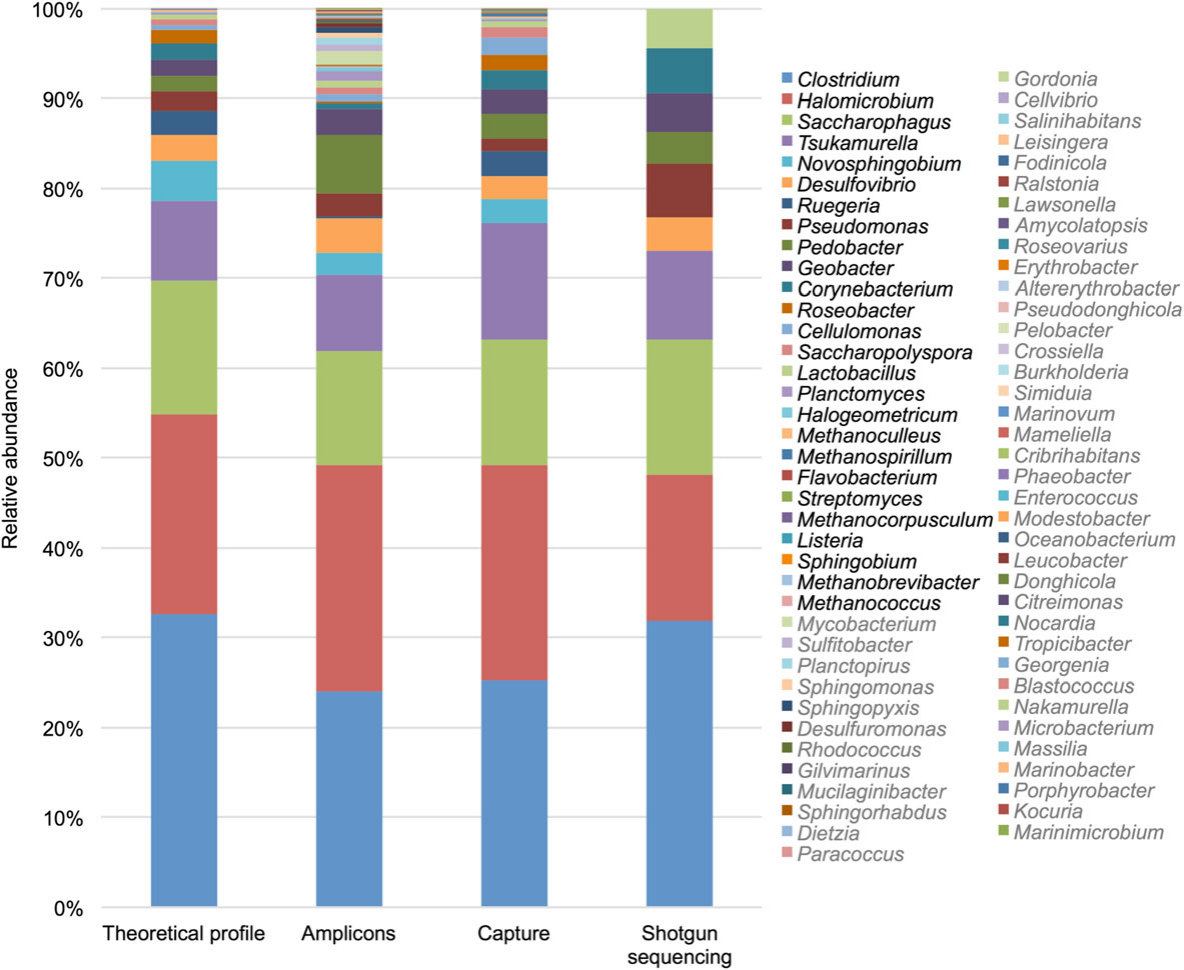
Microbial mock community profiles at the genus level for amplicon sequencing, hybridization capture, and shotgun sequencing. The genera identified through amplicon sequencing that are not part of the microbial mock community are indicated in gray. The abundance profiles at other taxonomic levels for the three datasets are available in Additional file 1: Figure S2

We then characterized the community structures obtained using the three methods through mapping of the reads against the 16S rDNA sequences of the Silva database. In this way, we showed that with hybridization capture, 24 of the 26 genera are detected and that the 16S reference sequences of these genera are covered over their entire length with a minimum coverage of 180X (Fig. 1). The 24 genera detected, ranging from 32.63% (*Clostridium)* to 0.00059% (*Sphingobium*) of the community, demonstrate the ability of the method to detect rare microorganisms. Thus, only the two less abundant species, *Methanobrevibacter smithii* and *Methanococcus aeolicus* (0.00006% each), are missed by hybridization capture with this sequencing depth (1,097,064 pairs of reads). However, when mapping the hybridization capture reads prior to their normalization (9,259,211 pairs of reads), *Methanobrevibacter smithii* (0.00006%) is also detected in the sample bringing to 25 out of 26 the number of genera detected and decreasing the detection limit to 0.00006%. With shotgun sequencing, only 10 dominant genera are identified in the dataset because of the difficulty of detecting the less abundant species in metagenomic samples even at high sequencing depths (Fig. 1). Finally, with amplicon sequencing, 23 out of the 26 genera are detected (32.63 to 0.00059% of the community), but contrary to hybridization capture and shotgun sequencing, this method also detects 49 other genera (ranging from 1.50 to 0.01% of the community) that are not part of the mock community (Fig. 1). This overestimation of diversity can be explained by the inaccurate assignment of the short fragments amplified by the primer pair, leading to a biased description of communities. Finally, by covering the complete 16S rDNA sequences, reads obtained through hybridization capture allow an accurate assignment of sequences up to the species and most of the time up to the strain level. In this way, hybridization capture clearly distinguishes the different species in the mock community contrary to amplicon sequencing that overestimates the diversity at low-resolution taxonomic levels. As a consequence, at the genus level but also at all the other taxonomic ranks, the description of community differs between the three methods (Fig. 1, Additional file 1: Figure S2). Indeed, in addition to detecting nearly all of the species present in the sample, hybridization capture yields abundances for the different species close to that expected (Additional file 1: Table S1). Even if slight variations of abundance tending to overestimate the rare species appear between the theoretical and hybridization capture profiles (1.63 ± 2.13% when abundance is ≥ 1%; 0.14 ± 0.18% when abundance is ≤ 1%), the relative abundance of species in the community is well estimated.

Thus, the use of hybridization capture on this microbial mock community demonstrates that the method gives a vision very close to the expected community structure with a high sensitivity, contrary to shotgun sequencing that gives a description restricted to the dominant species and to amplicon sequencing that does not allow an accurate assignment of reads and gives a biased vision of diversity. Further hybridization capture experiments could be carried out on the same microbial mock community to assess the reproducibility of the method. However, the high reproducibility of hybridization capture experiments performed on other biological models such as human exome [33–35] has already been demonstrated.

### Soil microbial community profiling

We then applied our hybridization capture method to a soil sample, considered as the most complex metagenomic sample, to demonstrate its efficiency on real microbial communities. As previously, we first estimated the proportion of reads reflecting 16S rRNA genes sequenced using the different approaches and demonstrated that hybridization capture also leads to a significant enrichment of the targeted biomarker in this complex sample. Indeed, with hybridization capture, the 16S rRNA gene represents 58.74% of the reads compared with shotgun sequencing, in which only 0.09% of sequences carry the biomarker. Even if significant and sufficient for the description of communities, this enrichment is probably less important than the enrichment obtained with the mock community because of the bigger complexity of this soil sample.

**Fig. 2:**
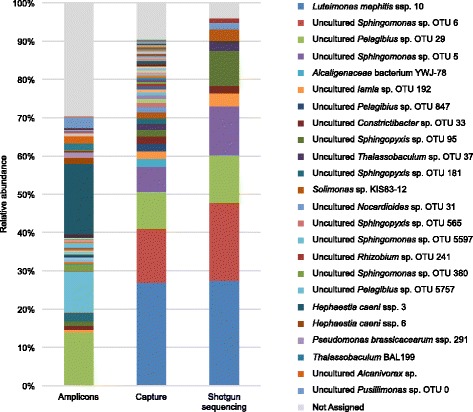
Soil composition profiles at the species level for amplicon sequencing, hybridization capture, and shotgun sequencing. Only the dominant bacterial species (relative abundance > 1%) are indicated in the legend. The abundance profiles at other taxonomic levels for the three datasets are available in Additional file 1: Figure S3

We then conducted operational taxonomic unit (OTU) reconstruction for the 16S rRNA genes from the three datasets using the dedicated EMIRGE software [11] and classified them with the Silva [28] database after chimera detection. At all taxonomic ranks, from domain to species, the community structures differed between the three methods (Fig. 2, Additional file 1: Figure S3). Indeed, 354 OTUs representing species were identified through capture compared with 13 OTUs through shotgun sequencing and 115 OTUs through amplicon sequencing. The lack of OTUs identified by shotgun sequencing is likely attributable to the complexity of the soil ecosystem and the difficulty of obtaining exhaustive 16S rRNA gene sequences even at very high sequencing depths. Similarly, amplicon sequencing is limited by the inability of “universal” primer pairs to target all the diversity of the 16S rRNA genes and by the possible inefficient amplification of complex metagenomic samples (Fig. 2, Table 1).

**Table 1:** Classification of 16S rDNA sequences into different taxonomic levels. Numbers in parentheses indicate the number of corresponding operational taxonomic units

	Hybridization capture		Amplicons		Shotgun sequencing	
Taxonomic level	No. of taxa	% unassigned sequences	No. of taxa	% unassigned sequences	No. of taxa	% unassigned sequences
Domain	2 (354)	0 (0)	1 (115)	0 (0)	1 (13)	0 (0)
Phylum	18 (353)	0.007 (1)	8 (115)	0 (0)	3 (13)	0 (0)
Class	42 (352)	0.014 (2)	16 (115)	0 (0)	5 (13)	0 (0)
Order	73 (338)	0.284 (16)	29 (114)	0.295 (1)	8 (13)	0 (0)
Family	117 (307)	1.260 (47)	35 (112)	0.543 (3)	9 (13)	0 (0)
Genus	143 (263)	4.600 (91)	48 (100)	11.797 (15)	12 (13)	0 (0)
Species	190 (190)	9.419 (164)	73 (73)	29.464 (42)	11 (11)	4.091 (2)

We observed that with hybridization capture, bacteria (99.63% of the 16S rDNA reads) and archaea (0.37% of the 16S rDNA reads) were simultaneously revealed, in contrast to the other approaches (Additional file 1: Figure S3a), even though the primer pair used for 16S amplicon sequencing also targets archaea [36] as demonstrated when studying the mock community. Archaea are relatively rare in soil, with Thaumarchaeota being the dominant archaeal group [37], as we observed by gene capture, revealing the sensitivity of this approach. Despite the differences in the structures of the microbial communities identified between the three methods, the same environmental taxa dominate the three datasets at low-resolution classification (Additional file 1: Figure S3). Proteobacteria, known to be abundant in soil [37] and to comprise many HCH-degrading species, form the dominant phylum in the three samples, representing 92.09, 89.53, and 93.44% of the reads by amplicon sequencing, hybridization capture, and shotgun sequencing, respectively. The Proteobacteria were primarily represented by Alphaproteobacteria (65.97, 53.24, and 62.94% of the reads, respectively) and Gammaproteobacteria (20.95, 32.35, and 30.50% of the reads, respectively) (Additional file 1: Figure S3b, c). In regard to the family level, Sphingomonadaceae (30.91 to 42.45% of the reads), Xanthomonadaceae (13.56 to 29.78% of the reads), and Rhodospirillaceae (14.93 to 18.40% of the reads) dominate the three datasets (Additional file 1: Figure S3e). However, at the genus level, 16S rRNA gene amplicon sequencing almost failed to detect *Luteimonas* (0.20% of the reads in contrast to 27.35 and 27.99% of the reads through shotgun sequencing and hybridization capture, respectively), one of the three dominant taxa of *Sphingomonas* (24.14 to 33.36% of the reads), and *Pelagibius* (12.21 to 15.47% of the reads) identified through hybridization capture and shotgun sequencing (Additional file 1: Figure S3f), thus generating a different profile of the microbial community at the species level (Fig. 2). Indeed, the dominant species identified through hybridization capture and shotgun sequencing, *Luteimonas mephitis* ssp. 10 (26.81 and 27.26% of the reads, respectively) and uncultured *Sphingomonas* sp. OTU 6 (14.21 and 20.52% of the reads, respectively), were missed by amplicon sequencing, which instead includes among its dominant species *Hephaestia caeni* ssp. 3 (18.39% of the reads), a species absent in the two other sequencing datasets. Consequently, whereas all 11 species identified through shotgun sequencing correspond to the dominant species identified using hybridization capture, only 30 of the 73 species identified with amplicon sequencing are shared with those obtained using hybridization capture (Table 1). This difference is most likely due to the inaccurate classification of short rDNA regions from amplicons at such a precise taxonomic level. These results highlight that hybridization capture is able to reveal diversity that is missed using the other two methods.

Another major difference that appears between the three approaches is the percentage of unassigned 16S rDNA sequences at the level of species: 4.09, 9.42, and 29.46%, respectively, for shotgun sequencing, hybridization capture, and PCR sequencing (Fig. 2, Table 1). For PCR sequencing, because of the short amplicon size (approximately 300 bp), accurate phylogenetic assignment of sequences is possible down to the taxonomic order or family but is unachievable at the genus level for most of sequences. By contrast, hybridization capture and shotgun sequencing allow for the reconstruction of complete or almost complete (1385 ± 121 and 1239 ± 253 bp, respectively) 16S rRNA genes, thereby facilitating accurate taxonomic classification. However, even with a full-length 16S rRNA gene, OTUs remain unassigned with hybridization capture, demonstrating its capability of revealing novel 16S rDNA sequences not described in current databases.

### Discovery of new taxa

**Fig. 3:**
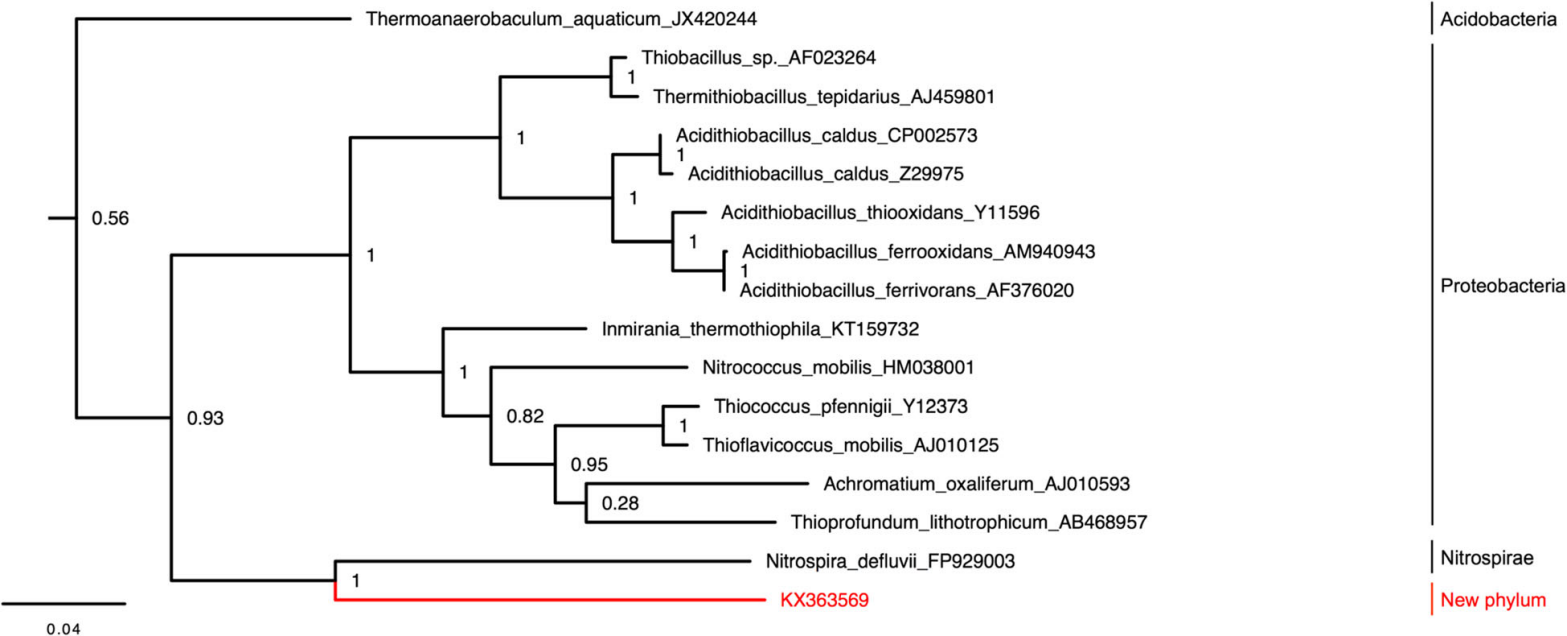
Novel candidate phylum position in a 16S rDNA maximum likelihood tree. The candidate phylum is represented in red. The names for the representative species, their accession numbers, and their phyla are given. The numbers at the nodes indicate the branch support calculated with the Shimodaira–Hasegawa test. The scale bar indicates 4% sequence divergence. The complete tree with the 50 sequences used is available in Additional file 1: Figure S4

To further demonstrate the capability of hybridization capture to reveal novel sequences, we analyzed the unassigned 16S rDNA sequences at low-resolution taxonomic levels through the placement of the sequences into phylogenetic trees. Analysis of a full-length 16S rDNA sequence not assigned at the level of phylum revealed that the sequence forms a discrete clade (Fig. 3, Additional file 1: Figure S4a) close to the Nitrospirae. Alignment of the 16S rRNA gene with the closest sequences (Additional file 1: Figure S4b) based on this phylogeny and an exhaustive environmental phylogeny [38] revealed that the unidentified 16S rDNA sequence was less than 80% identical to the other sequences, suggesting that this 16S rRNA gene belongs to a new candidate phylum. Similarity searches in nr database from NCBI revealed two identical environmental sequences (JN607053.1, DQ499315.1) identified as “uncultured bacterium” isolated from microorganisms from two different cave soils. We also reanalyzed the unassigned 16S rDNA sequences for hierarchical class and order and determined their probable classifications to new taxa. Indeed, we assigned one sequence attributed to the Gemmatimonadetes phylum to a new class (Additional file 1: Figure S5), another sequence to a new class belonging to the Chloroflexi (Additional file 1: Figure S6), and nine new sequences to two Saccharibacteria (Candidate Division TM7) orders (Additional file 1: Figure S7).

## Conclusions

Here, we demonstrate that hybridization capture targeting the rRNA gene represents a promising approach for microbial community studies. Through the significant enrichment of the 16S rRNA gene (up to 97.30% of reads), hybridization capture gives an accurate description of communities compared to other conventional molecular methods. Indeed, as evidenced by our results, 16S rRNA gene amplicon sequencing and shotgun sequencing missed taxa, regardless of their abundance in the sample, leading to different community structure descriptions at the genus and species levels. Hybridization capture correlates with conventional approaches at low-resolution taxonomic classification but reveals a broader microbial diversity with its capacity to target even the least abundant species through efficient 16S rRNA gene enrichment. Indeed, based on our experiments, hybridization capture is able to reveal microorganisms that only represent 0.00006% of the community. Therefore, because of its sensitivity and the use of explorative probes, this hybridization capture method enables the identification of 16S rRNA genes belonging to new taxa, allowing the discovery of novelty from the phylum to the species level. Significantly increasing the sequencing depths of shotgun and amplicon sequencing could reveal greater 16S rRNA gene diversity. Nevertheless, for the latter, because of PCR biases, all taxa will never be amplified commensurately, and artificial diversity can be created. Moreover, due to the small sizes of the sequences generated, accurate phylogenetic assignment of reads is infeasible at high-resolution taxonomic levels. By contrast, hybridization capture allows for the reconstruction of complete 16S rRNA gene sequences that are needed to accurately confer phylogenetic associations [39, 40]. Hybridization capture targeting rRNA genes could consequently represent an innovative strategy to describe microbial community structure.

## Additional file

Additional file 1: **Figure S1.** Schematic representation of the hybridization capture method. **Figure S2.** Mock community profiles at different taxonomic levels for 16S rRNA gene amplicon sequencing, hybridization capture, and shotgun sequencing. **Figure S3.** Soil prokaryote composition profiles at different taxonomic levels for 16S rRNA gene amplicon sequencing, hybridization capture, and shotgun sequencing. **Figure S4.** Phylogenetic position of an unassigned sequence to a new phylum. **Figure S5.** Phylogenetic position of an unassigned sequence to a new class belonging to the Gemmatimonadetes phylum. **Figure S6.** Phylogenetic position of an unassigned sequence to a new class belonging to the Chloroflexi phylum. **Figure S7.** Phylogenetic position of new unassigned sequences to the Saccharibacteria phylum. **Table S1.** Microbial mock community used for hybridization capture validation and 16S rDNA relative abundances observed using the three methods (amplicons, capture, and shotgun sequencing). **Table S2.** Set of probes targeting the 16S rRNA gene used for hybridization capture. (DOCX 4852 kb)
