# Supplementary material for: Hybridization capture reveals microbial diversity missed using current profiling methods
Source: Microbiome. 2018 Mar 27;6:61. doi: 10.1186/s40168-018-0442-3 (PMC5870382; doi:10.1186/s40168-018-0442-3)
Supplement: Supplementary file 1 — Figure S1. Schematic representation of the hybridization capture method. Figure S2. Mock community profiles at different taxonomic levels for 16S rRNA gene amplicon sequencing, hybridization capture, and shotgun sequencing. Figure S3. Soil prokaryote composition profiles at different taxonomic levels for 16S rRNA gene amplicon sequencing, hybridization capture, and shotgun sequencing. Figure S4. Phylogenetic position of an unassigned sequence to a new phylum. Figure S5. Phylogenetic position of an unassigned sequence to a new class belonging to the Gemmatimonadetes phylum. Figure S6. Phylogenetic position of an unassigned sequence to a new class belonging to the Chloroflexi phylum. Figure S7. Phylogenetic position of new unassigned sequences to the Saccharibacteria phylum. Table S1. Microbial mock community used for hybridization capture validation and 16S rDNA relative abundances observed using the three methods (amplicons, capture, and shotgun sequencing). Table S2. Set of probes targeting the 16S rRNA gene used for hybridization capture. (DOCX 4852 kb) [file 40168_2018_442_MOESM1_ESM.docx]

**Hybridization capture reveals microbial diversity missed using current profiling methods**

Cyrielle Gasc^1^ and Pierre Peyret^1*^

^1^ Université Clermont Auvergne. INRA. MEDIS. F-63000 Clermont-Ferrand. France

* Correspondence : pierre.peyret@uca.fr

**Table of contents:**

**Figure S1** Schematic representation of the hybridization capture method.

**Figure S2** Mock community profiles at different taxonomic levels for 16S rRNA gene amplicon sequencing. hybridization capture and shotgun sequencing.

**Figure S3** Soil prokaryote composition profiles at different taxonomic levels for 16S rRNA gene amplicon sequencing. hybridization capture and shotgun sequencing.

**Figure S4** Phylogenetic position of an unassigned sequence to a new phylum.

**Figure S5** Phylogenetic position of an unassigned sequence to a new class belonging to the Gemmatimonadetes phylum.

**Figure S6** Phylogenetic position of an unassigned sequence to a new class belonging to the Chloroflexi phylum.

**Figure S7** Phylogenetic position of new unassigned sequences to the Saccharibacteria phylum.

**Table S1** Microbial mock community used for hybridization capture validation and 16S rDNA relative abundances observed using the three methods (amplicons, capture and shotgun sequencing).

**Table S2** Set of probes targeting the 16S rRNA gene used for hybridization capture.

**Figure S1** Schematic representation of the hybridization capture method. A sequencing library containing the targeted biomarker is constructed and hybridized in solution against a set of biotinylated specific probes. Probe-DNA heteroduplexes are captured with streptavidin-coated magnetic beads. and non-target sequences are washed away. The enriched sample is eluted. amplified and subjected to a second round of capture before sequencing.

**Figure S2** Mock community profiles at different taxonomic levels for 16S rRNA gene amplicon sequencing. hybridization capture and shotgun sequencing. a. Domain. b. Phylum. c. Class. d. Order. e. Family.

**Figure S3** Soil prokaryote composition profiles at different taxonomic levels for 16S rRNA gene amplicon sequencing. hybridization capture and shotgun sequencing. a. Domain. b. Phylum. c. Class. d. Order. e. Family. f. Genus. Only the dominant orders. families and genera (relative abundance > 0.1%) are indicated in the legend. Unc = Uncultured. Grp = Group.

**a**

**b**

**Figure S4** Phylogenetic position of an unassigned sequence to a new phylum. a. Novel phylum position in a 16S rDNA maximum likelihood tree. The new phylum is represented in red. The names for the representative species. their accession numbers and their phyla are given. The numbers at the nodes indicate the branch support calculated with the Shimodaira-Hasegawa test. The scale bar indicates 4% sequence divergence. b. Matrix of pairwise genetic distances between the unassigned phylum sequence and its closest reference sequences based on the 16S rRNA gene. The first two columns indicate representative sequences assignment and accession numbers. Sequences 2 to 12 are the closest sequences based on Figure 2. Sequences 13 to 17 are the closest sequences based on an exhaustive environmental phylogeny [1]. The threshold for phylum determination is set at 80%.

**Figure S5** Phylogenetic position of an unassigned sequence to a new class belonging to the Gemmatimonadetes phylum. a. Novel class position in a 16S rDNA maximum likelihood tree. The new class is represented in red. The names for the representative species. their accession numbers and their phyla are given. The numbers at the nodes indicate the branch support calculated with the Shimodaira-Hasegawa test. The scale bar indicates 3% sequence divergence. b. Matrix of pairwise genetic distances between the unassigned class sequence and its closest reference sequences based on the 16S rRNA gene. The first two columns indicate representative sequences assignment and accession numbers. Sequences are the closest sequences based on the tree. The threshold for class determination is set at 85%.

**Figure S6** Phylogenetic position of an unassigned sequence to a new class belonging to the Chloroflexi phylum. a. Novel class position in a 16S rDNA maximum likelihood tree. The new class is represented in red. The names for the representative species. their accession numbers and their phyla are given. The numbers at the nodes indicate the branch support calculated with the Shimodaira-Hasegawa test. The scale bar indicates 3% sequence divergence. b. Matrix of pairwise genetic distances between the unassigned class sequence and its closest reference sequences based on the 16S rRNA gene. The first two columns indicate representative sequences assignment and accession numbers. Sequences are the closest sequences based on the tree. The threshold for class determination is set at 85%.

**Figure S7** Phylogenetic position of new unassigned sequences to the Saccharibacteria phylum. a. Novel sequences position in a 16S rDNA maximum likelihood tree. The new sequences forming two orders are represented in red. The names for the representative species. their accession numbers and their phyla are given. The numbers at the nodes indicate the branch support calculated with the Shimodaira-Hasegawa test. The scale bar indicates 4% sequence divergence. b. Matrix of pairwise genetic distances between the unassigned sequences and their closest reference sequences based on the 16S rRNA gene. The first two columns indicate representative sequences assignment and accession numbers. Sequences are the closest sequences based on the tree. The threshold for order determination is set at 85%.

**Table S1** Microbial mock community used for hybridization capture validation and 16S rDNA relative abundances observed using the three methods (amplicons, capture and shotgun sequencing).

|  | **Species** | **Domain** | **DSMZ number** | **Theoretical**  **16S rDNA**  **relative abundance (%)** | **Amplicons**  **(%)** | **Capture**  **(%)** | **Shotgun sequencing**  **(%)** |
| --- | --- | --- | --- | --- | --- | --- | --- |
| 1 | *Clostridium acetobutylicum* | Bacteria | 792 | 32.63011 | 24.06882 | 25.21885 | 31.89891 |
| 2 | *Halomicrobium mukohataei* | Archaea | 12286 | 22.24780 | 25.10281 | 23.94701 | 16.23975 |
| 3 | *Saccharophagus degradans* | Bacteria | 17024 | 14.83187 | 12.74913 | 13.97188 | 15.09563 |
| 4 | *Tsukamurella paurometabola* | Bacteria | 20162 | 8.89912 | 8.42835 | 13.03462 | 9.83607 |
| 5 | *Novosphingobium pentaromativorans* | Bacteria | 17173 | 4.44956 | 2.49003 | 2.59445 | 0 |
| 6 | *Desulfovibrio vulgaris* | Bacteria | 644 | 2.96637 | 3.84861 | 2.56435 | 3.73975 |
| 7 | *Ruegeria pomeroyi* | Bacteria | 15171 | 2.66974 | 0.15290 | 2.77102 | 0 |
| 8 | *Pseudomonas putida* | Bacteria | 6125 | 2.07646 | 2.61392 | 1.42153 | 5.92555 |
| 9 | *Corynebacterium glutamicum* | Bacteria | 20300 | 1.77982 | 0.66319 | 2.20026 | 5.00342 |
| 10 | *Geobacter lovleyi* | Bacteria | 17278 | 1.77982 | 2.89216 | 2.57344 | 4.25205 |
| 11 | *Pedobacter heparinus* | Bacteria | 2366 | 1.77982 | 6.47980 | 2.85448 | 3.58607 |
| 12 | *Roseobacter denitrificans* | Bacteria | 7001 | 1.48319 | 0.26174 | 1.65505 | 0 |
| 13 | *Cellulomonas flavigena* | Bacteria | 20109 | 0.59327 | 0.75315 | 1.95778 | 0 |
| 14 | *Saccharopolyspora erythraea* | Bacteria | 40517 | 0.59327 | 0.74301 | 1.22774 | 0 |
| 15 | *Lactobacillus delbrueckii* | Bacteria | 20081 | 0.53395 | 0.71757 | 0.58578 | 4.42281 |
| 16 | *Halogeometricum borinquense* | Archaea | 11551 | 0.17798 | 0.42601 | 0.19895 | 0 |
| 17 | *Planctomyces limnophilus* | Bacteria | 3776 | 0.17798 | 1.04254 | 0.13856 | 0 |
| 18 | *Methanoculleus marisnigri* | Archaea | 1498 | 0.14832 | 0.08217 | 0.16920 | 0 |
| 19 | *Methanospirillum hungateii* | Archaea | 864 | 0.11865 | 0.10069 | 0.28483 | 0 |
| 20 | *Lactobacillus brevis* | Bacteria | 20054 | 0.02076 | 0.01265 | 0.04365 | 0 |
| 21 | *Flavobacterium psychrophilum* | Bacteria | 21280 | 0.01780 | 0.01433 | 0.19447 | 0 |
| 22 | *Streptomyces avermitilis* | Bacteria | 46492 | 0.01068 | 0.01223 | 0.18091 | 0 |
| 23 | *Methanocorpusculum labreanum* | Archaea | 4855 | 0.00890 | 0.01182 | 0.07954 | 0 |
| 24 | *Listeria welshimeri* | Bacteria | 20650 | 0.00356 | 0 | 0.06659 | 0 |
| 25 | *Sphingobium indicum* | Bacteria | 16412 | 0.00059 | 0.07103 | 0.06384 | 0 |
| 26 | *Clostridium leptum* | Bacteria | 753 | 0.00047 | 0 | 0.00121 | 0 |
| 27 | *Methanobrevibacter smithii* | Archaea | 861 | 0.00006 | 0 | 0 | 0 |
| 28 | *Methanococcus aeolicus* | Archaea | 17508 | 0.00006 | 0 | 0 | 0 |

**Table S2** Set of probes targeting the 16S rRNA gene used for hybridization capture.

| **Probe name** | **Sequence (location on rRNA gene)** | **Length (bases)** | **Degeneracy** | **Melting temperature (°C)** | **Location on**  **16S rRNA gene (bases)** |
| --- | --- | --- | --- | --- | --- |
| 16S_1 | CCAGACTCCTACGGGAGGCAGCAGTGGGGAA | 31 | 1 | 69.7 | 300 |
| 16S_2 | AAACTCCTACGGGAGGCAGCAGTGGGGAATCT | 32 | 1 | 67.0 | 350 |
| 16S_3 | CRAACSGGATTAGATACCCSGGTAGTCC | 28 | 8 | 62.9 – 64.3 | 700 |
| 16S_4 | AACAGGATTAGATACCCTGGTAGTCCACGCC | 31 | 1 | 64.4 | 750 |
| 16S_5 | GGGAGCAAACAGGATTAGATACCCTGGTAGT | 31 | 1 | 63.0 | 750 |
| 16S_6 | AACAGGATTAGATACCYTGGYAGTCCACGC | 30 | 4 | 61.6 – 64.4 | 800 |
| 16S_7 | AACAGGATWAGATACCCKGGYAGTCCAYRC | 30 | 32 | 60.3 – 65.7 | 800 |
| 16S_8 | ACTCAAAGGAATTGACGGGGGCCCGCACAAG | 31 | 1 | 67.0 | 850 |
| 16S_9 | CACAAGCGGTGGAGCATGTGGTTTAATTCGA | 31 | 1 | 63.0 | 900 |
| 16S_10 | CGCAAGDRTGAAACTTAAAGGAATTGGCGGGGGAGCAC | 38 | 6 | 67.7 – 69.9 | 900 |
| 16S_11 | GTTGGGTTAAGTCCCGCAACGAGCGCAACCC | 31 | 1 | 68.3 | 1000 |
| 16S_12 | GAGAGGWGGTGCATGGCCGYCGYCAGYTCGT | 31 | 16 | 68.3 – 72.3 | 1000 |
| 16S_13 | CATGGTTGTCGTCAGCTCGTGTCGTGAGATG | 31 | 1 | 65.7 | 1100 |
| 16S_14 | TGTCGTCAGCTCGTGTCGTGAGATGTTGGGTTAAGTCCCGCAACGAGCSS | 50 | 4 | 75.2 | 1100 |
| 16S_15 | TCGTCAGCTCGTGTYGTGAGRTGTTSGGTTAAGTCC | 36 | 8 | 66.7 – 69.0 | 1100 |

**References**

1. Hug LA. Baker BJ. Anantharaman K. Brown CT. Probst AJ. Castelle CJ. Butterfield CN. Hernsdorf AW. Amano Y. Ise K. et al: A new view of the tree of life. *Nat Microbiol* 2016. 1:16048.
